# *TimiRGeN*: *R/Bioconductor* package for time series microRNA–mRNA integration and analysis

**DOI:** 10.1093/bioinformatics/btab377

**Published:** 2021-05-16

**Authors:** K Patel, S Chandrasegaran, I M Clark, C J Proctor, D A Young, D P Shanley

**Affiliations:** Campus for Ageing and Vitality, Biosciences Institute, Newcastle University, Newcastle upon Tyne NE4 5PL, UK; Campus for Ageing and Vitality, Biosciences Institute, Newcastle University, Newcastle upon Tyne NE4 5PL, UK; School of Biological Sciences, University of East Anglia, Norwich NR4 7TJ, UK; Campus for Ageing and Vitality, Biosciences Institute, Newcastle University, Newcastle upon Tyne NE4 5PL, UK; Life Science Centre, Biosciences Institute, Newcastle University, Newcastle upon Tyne NE1 4EP, UK; Campus for Ageing and Vitality, Biosciences Institute, Newcastle University, Newcastle upon Tyne NE4 5PL, UK

## Abstract

**Motivation:**

The analysis of longitudinal datasets and construction of gene regulatory networks (GRNs) provide a valuable means to disentangle the complexity of microRNA (miRNA)–mRNA interactions. However, there are no computational tools that can integrate, conduct functional analysis and generate detailed networks from longitudinal miRNA–mRNA datasets.

**Results:**

We present *TimiRGeN*, an *R* package that uses time point-based differential expression results to identify miRNA–mRNA interactions influencing signaling pathways of interest. miRNA–mRNA interactions can be visualized in *R* or exported to *PathVisio* or *Cytoscape*. The output can be used for hypothesis generation and directing *in vitro* or further *in silico* work such as GRN construction.

**Availability and implementation:**

*TimiRGeN* is available for download on Bioconductor (https://bioconductor.org/packages/TimiRGeN) and requires *R* v4.0.2 or newer and *BiocManager* v3.12 or newer.

**Supplementary information:**

Supplementary data are available at *Bioinformatics* online.

## 1 Introduction

MicroRNAs (miRNAs) are single-stranded functional RNAs, around 16–22 nucleotides long which target specific mRNAs for degradation or translational repression; thus affecting protein levels (Selbach *et al.*, 2008). Targeting is achieved by complementary binding between the 3ʹUTR of the target mRNA and a 7–8 nucleotide sequence found on the 5ʹend of the miRNA, known as the seed sequence (Bartel, 2004). There is increased clinical interest in miRNAs for several reasons: (i) miRNAs can be tested in animal models to understand human diseases and conditions. An example is miR-140-5p which is upregulated during chondrogenesis and downregulated during osteoarthritis (Barter *et al.*, 2015; Miyaki *et al.*, 2010). (ii) miRNAs can be secreted via exosomes into surrounding blood, extracellular matrix and urine (Chaturvedi *et al.*, 2015; Chen *et al.*, 2017; Leidinger *et al.*, 2013). Their presence in body fluids provides valuable noninvasive biomarkers to assess the state of difficult to access tissues such as tumors, brain and bone. (iii) Lastly, miRNAs have potential as therapeutic agents as they modulate expression of specific mRNAs (Schwarzenbach *et al.*, 2014).

However, in the laboratory, miRNAs are difficult to study, primarily because a single miRNA can regulate many mRNAs and a single mRNA can be regulated by multiple miRNAs. miRNA–mRNA interactome studies report over 18 000 interactions in HEK293 cells and over 34 000 interactions in human hepatoma cells (Helwak *et al.*, 2013; Moore *et al.*, 2015). A complementary strategy is to use a computational approach. The analysis of longitudinal miRNA–mRNA expression data, construction of gene regulatory networks (GRNs) and subsequent dynamic modeling, is a particularly useful means to gain a better understanding of miRNA–mRNA interactions (Ooi *et al.*, 2018; Proctor *et al.*, 2017; Qin *et al.*, 2015). GRNs are useful tools for integrating multiomic data on mechanistic schematics. Yet, currently there is no computational tool that can handle longitudinal miRNA–mRNA datasets and reduce the volume of data to an extent where GRN construction is possible. This is presented in Table 1.

**Table 1.** miRNA–mRNA integration tools

Tool name	Availability	Time	Funct analysis	Reduction	Updated
*anamiR*	Bioc	X	✓:Kegg, React,+	✓	2018
*DREM2*	Install	✓	✓:GO	X	2020
*MAGIA2*	Online	X	✓:DAVID	✓	2012
*miARMa-seq*	Install	✓	✓:GO, Kegg	X	2019
*miRComb*	SF	✓	✓:GO, Kegg	✓	2020
*miRIntegrator*	Bioc	X	✓:Kegg, React	✓	2016
*miRNet*	Online	X	✓:GO, Kegg	X	2021
*miRTarVis+*	Online	X	X	✓	2020
*Sigterms*	SF	X	✓:GO	✓	2009
*SpidermiR*	Bioc	X	X	✓	2020
*ToppMiR*	Online	X	✓:GO	✓	2021

*Note*: Comparison of miRNA–mRNA integration tools: several tools are available as *R* packages that can be downloaded from Bioc (Bioconductor) or SF (SourceForge). Other tools can be installed locally or are available online. Some tools are capable of handling time series datasets. Several can perform funct (functional) analysis, usually utilizing GO, Kegg, React (Reactome), DAVID or others (+) and a few tools can reduce the volume of data. Also shown is when each tool was last updated.

Many existing tools (Table 1) have particular strengths, but none satisfy the criteria necessary to bridge longitudinal multiomic data and GRN creation. *anamiR*, *miRIntegrator*, *MAGIA2*, *Sigterms* and *SpidermiR* have substantial miRNA–mRNA integration capabilities but cannot handle longitudinal datasets (Bisognin *et al.*, 2012; Cava *et al.*, 2017; Creighton *et al.*, 2008; Diaz *et al.*, 2017; Wang *et al.*, 2019). Web-based tools such as *miRNet*, *miRTarVis+* and *ToppmiR* have excellent visualization capabilities but also cannot analyze longitudinal datasets (Fan and Xia, 2018; L’Yi *et al.*, 2017; Wu *et al.*, 2014). *DREM2* and *miARMa-seq* handle longitudinal datasets, but do not reduce the volume of data enough for GRN generation (Andrés-León *et al.*, 2016; Schulz *et al.*, 2012). *miRComb* can use longitudinal data to generate miRNA–mRNA interactions networks, but the networks lack detail on upstream or downstream information, making the output insufficient for GRN generation (Vila-Casadesús *et al.*, 2016). Furthermore, several tools have not been actively maintained so their usability may be diminished.

**Fig. 1.**
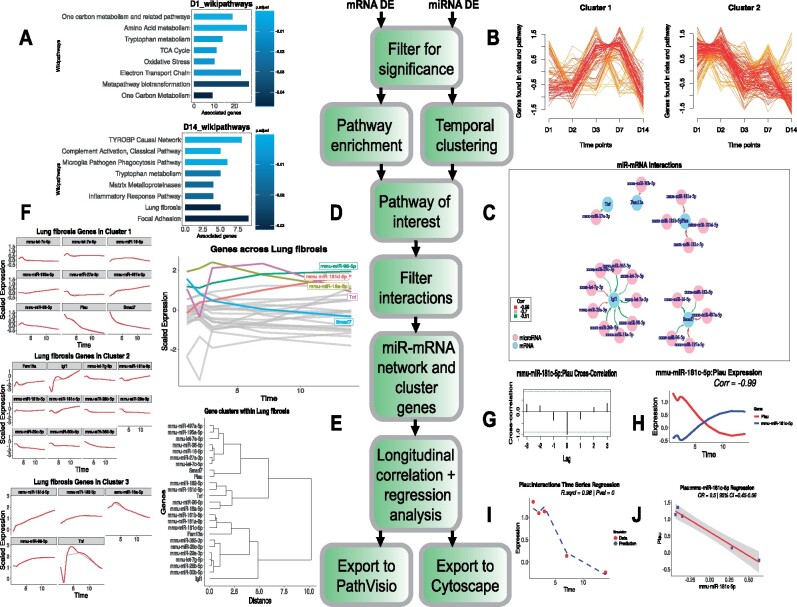
Pipeline of the *TimiRGeN R* package: The FA miRNA–mRNA data are input and filtered for significantly expressed genes for each time point. From here, one of two methods can be used to find WikiPathways of interest. (**A**) Time-dependent pathway enrichment to find enriched pathways at each time point. The enriched pathways are ranked in descending order of adjusted *P*-values on bar plots. Results from day 1 and day 14 are shown. Or (**B**) temporal clustering where global trends of the pathways over time are clustered. Two clusters are shown here. Each line is a pathway and the color represents how well a pathway fits into a cluster. Ranking from highest to lowest are: red, orange, yellow. miRNA–mRNA interactions within a selected signaling pathway can be predicted by filtration of miRNA–mRNA pairs using databases and correlation. (**C**) Filtered miRNA–mRNA pairs can be viewed in *R*. Nodes are pink for miRNAs or blue for mRNAs and edges are color coded by correlation over time. (**D**) Behavior of genes within the miRNA–mRNA interaction network can be viewed across the time course and genes which pass a threshold (>1.5 in this example) are highlighted. (**E**) The genes can also be hierarchically clustered to identify trends. (**F**) Expression changes within the clusters can be plotted. These line plots include a gray line (data points) and a red line (smooth spline). (**G**) A selected miRNA–mRNA pair (*mmu-miR-181c-5p* and *Plau*) can be analyzed using cross-correlation analysis. (**H**) The selected mRNA (red) and miRNA (blue) can also be displayed over the time course. The data are scaled and interpolated over a spline and the correlation is displayed. (**I**) Regression analysis can be performed on a selected miRNA or mRNA. *Plau* was selected, so its expression over time is predicted based on the chosen miRNAs that target it. In this example *mmu-miR-181c-5p* is selected to predict the behavior of *Plau*. Expression values of *Plau* are displayed as red dots and the predicted expression of *Plau* is displayed as a dashed blue line. *R*^2^ and *P*-value are shown. (**J**) Regression can also be performed between a miRNA–mRNA pair. The OR (odds-ratio) between the two time series can be calculated, along with the 95% CI (confidence intervals). Correlation, *R*^2^, *P*-value, OR and CI are rounded to 2 decimal places. Network data can be exported to *PathVisio* or *Cytoscape*

There is clearly a need for a tool that can integrate, functionally analyze and generate detailed networks from longitudinal miRNA–mRNA datasets, which can then be used to identify GRNs. Here, we present the *R/Bioconductor* package *TimiRGeN*, which uses differential expression (DE) data as input to generate small miRNA–mRNA interaction networks. Results from *TimiRGeN* can be exported to *Cytoscape* or *PathVisio* for further bioinformatic analysis (Kutmon *et al.*, 2015; Smoot *et al.*, 2011). The *TimiRGeN* package thereby provides a much-needed means to generate hypotheses from longitudinal multiomic datasets. To demonstrate the capabilities of the package several datasets were analyzed (see Section 2), including a comprehensive RNAseq time series miRNA–mRNA folic acid (FA)-induced mouse kidney injury dataset (Fig. 1) (Craciun *et al.*, 2016; Pellegrini *et al.*, 2016).

## 2 Materials and methods

FA data from GSE61328 (miRNA) and GSE65267 (mRNA) were downloaded using the *fastqc-dump* function from *SRA toolkit* and fastq files were checked with *FastQC* (Andrews, 2010; Leinonen *et al.*, 2011). *Cutadapt* removed adapter sequences from miRNA fastq files, and then the trimmed fastq files were processed with *mir2deep* (*mapper*, *quantifier* and *miRDeep2* functions) to produce mature miRNA data which could be imported into *R* (Friedlander *et al.*, 2012; Martin, 2011). *Salmon quant* aligned and quantified the mRNA fastq files, and *tximport* imported the output of *Salmon* into *R* (Patro *et al.*, 2017; Soneson *et al.*, 2015). Mouse transcriptome GRCm38.cdna.all was indexed for miRNA processing with *Bowtie build* and mRNA processing with *Salmon index* (Cunningham *et al.*, 2019; Langmead and Salzberg, 2012). In *R*, *limma* was used for DE analysis (Ritchie *et al.*, 2015). The *makeContrasts* function performed time point-based DE. The zero time point was contrasted against each subsequent time point (1, 2, 3, 7 and 14 days after FA injection). Results were analyzed with the *TimiRGeN R* package. For the FA kidney injury dataset, the combined mode of analysis found the ‘Lung fibrosis’ WikiPathway (WP3632) to be consistently enriched during days 3, 7 and 14 of the time course. The ‘Lung fibrosis’ pathway was analyzed for potential miRNA–mRNA interactions. Twenty interactions were kept because they were found in at least two databases and had Pearson correlations lower than −0.5. Results were exported to create a dynamic miRNA integrated Lung fibrosis signaling pathway in *PathVisio*. *CellDesigner* was then used to create an SBML formatted GRN (Funahashi *et al.*, 2008). A second mouse kidney injury dataset generated by Unilateral Ureter Obstruction (UUO) was downloaded from GSE118340 (miRNA) and GSE118339 (mRNA) (Pavkovic *et al.*, 2017). UUO and FA datasets were processed and analyzed using the same methods. A 10 time point longitudinal miRNA–mRNA breast cancer dataset was downloaded and processed as is described in Supplementary  Data. This dataset underwent two separate analysis with *TimiRGeN*. Once where *DESeq2* was used for pairwise DE and a second time where *DESeq2* performed whole time course DE with the *LRT* method (Baran-Gale *et al.*, 2016; Love *et al.*, 2014). A microarray hypoxia dataset was downloaded from GSE47534 and also put through *TimiRGeN* analysis (Camps *et al.*, 2014). The *lumi* and *AgiMicroRna* packages were used for processing and *limma* for pairwise DE (Du *et al.*, 2008; López-Romero, 2011). Microarray platforms GPL6884 and GPL8227 were downloaded and gene IDs extracted to create a list of probes for enrichment analysis. Scripts and data for reproducibility are linked to in Supplementary  Data.

## 3 Results

### 3.1 Time point and miRNA specific analysis

Pairwise miRNA and mRNA DE data (Log2FC and adjusted *P*-values) from each time point can be used as input for *TimiRGeN*. The tool works on RNAseq and microarray data, and it has two modes of analysis. The combined mode analyses miRNA and mRNA data from the same time point together, and here each gene from a time point can be filtered for significance independent of all other time points. The separate mode analyses miRNA and mRNA data independent of each other. Separate mode analysis allows for a miRNA or mRNA from a time point to be filtered for significance independent of all other time points and gene types (miRNA or mRNA). *TimiRGeN* uses WikiPathways for functional analysis, and most are curated by either entrez gene IDs or ensemble gene IDs so *TimiRGeN* provides both for the user. Neither of these annotation types can distinguish between −3p or −5p miRNAs, thus *TimiRGeN* also provides adjusted IDs, in case a miRNA–mRNA interaction network is generated with both the −3p and −5p versions of a miRNA.

### 3.2 Filtering data with time-based functional analysis

*TimiRGeN* offers two functional analysis methods: time-dependent pathway enrichment and temporal pathway clustering analysis. Both use the *rWikiPathways* package an API for the WikiPathways database to find signaling pathways of interest (Slenter *et al.*, 2018).

#### 3.2.1 Time-dependent pathway enrichment method

Overrepresentation analysis from *clusterProfiler* is applied to time series data (Yu *et al.*, 2012). Hypergeometric tests are performed to contrast the number of genes found in common between each time point (after filtering for significantly differentially expressed genes) and each species specific WikiPathway. This produces a list of enriched pathways for each time point (Fig. 1A). Alternatively, if the separate mode of analysis is applied, enrichment analysis is performed for each time point per gene type. The background/universe used to perform overrepresentation analysis can be adjusted by the user e.g. probes in a microarray or all known genes within a cell type.

#### 3.2.2 Temporal pathway clustering method

Temporal pathway clustering (Fig. 1B) utilizes *Mfuzz* (Kumar and Futschik, 2007). Supervised soft clusters are created based on temporal patterns which stem from the number of genes found in each time point (after filtering for significance) and each species specific WikiPathway. This will show global trends within the dataset. Pathways are assigned fitness scores for each cluster, from 0 to 1, and these can be filtered to find highly correlating pathways in clusters of interest. If the separate mode is used, temporal pathway clustering is performed for each gene type individually.

### 3.3 Filter miRNA–mRNA interactions from a signaling pathway of interest

After a signaling pathway has been selected for further analysis, the *TimiRGeN* pipeline will extract each mRNA that is found in common between the selected pathway and the input mRNA data. Each of these mRNAs are assumed to be potential targets of every miRNA in the input data. This results in a miRNA–mRNA interaction matrix which can be used to filter out miRNA–mRNA interactions that are not likely to occur by using correlations and miRNA–mRNA interaction databases TargetScan, miRDB and miRTarBase (Agarwal *et al.*, 2015; Chen and Wang, 2020; Huang *et al.*, 2020). Correlations are calculated for changes over time (Log2fc or average expression) between a given miRNA and a given mRNA. The default method is Pearson, but users can also select between Spearman or Kendall. Since miRNAs negatively regulate mRNAs, highly negative correlation values from miRNA–mRNA pairs could be used to identify miRNA–mRNA interactions that are likely regulate the selected pathway. Users can define a correlation threshold to filter for miRNA–mRNA interactions. The default setting for maximum correlation is −0.5. Three miRNA target databases are also usable to filter for miRNA–mRNA interactions. This includes two predictive target databases (TargetScan and miRDB) and one functional database (miRTarBase) which has had all functional support labeled as ‘weak’ removed. Predictive databases TargetScan and miRDB were selected because, although they have differences in their prediction methods, they share usage of 3ʹUTR-seed site complementarity and seed site conservation to predict miRNA–mRNA interactions (Peterson *et al.*, 2014). Comparisons between different miRNA–mRNA prediction methods find that 3ʹUTR-seed site complementarity identify the most true positive miRNA–mRNA interactions (Mazière and Enright, 2007; Zhang and Verbeek, 2010). Interactions found or not found in the three databases will be represented as 1 or 0, respectively. Users have the option to choose which combination of databases they wish to mine information from and they can choose the number of databases which an interaction needs to be mined from to be filtered. The default setting for the minimum number of databases needed to filter a miRNA–mRNA interaction is 1. Once correlation and databases have been used to filter for miRNA–mRNA interactions which may be affecting the signaling pathway of interest, they can be displayed in an internal *R* network (Fig. 1C). Resulting genes found in the miRNA–mRNA interaction network can be viewed over the time course. Here, genes that pass a user-defined threshold can be highlighted (Fig. 1D). The genes can also be sorted into hierarchical clusters shown by a dendrogram, from which clusters can be plotted to show the behavior of the genes (Fig. 1E and F). A heatmap, which is compatible with the dendrogram, can also be generated (Supplementary Figure S1).

### 3.4 Longitudinal miRNA–mRNA pair analysis

The *TimiRGeN R* package has a suite of longitudinal analysis approaches for analyzing predicted miRNA–mRNA interacting pairs. This includes several correlation- and regression-based methods which are commonly used to analyze longitudinal datasets (Ding and Bar-Joseph, 2020). Cross-correlation analysis is a useful method to determine similarity between two time series (Fig. 1G). If the time series is of sufficient length, the metric can be used to identify delays and further filter for miRNA–mRNA interacting pairs with interesting dynamics (Jung *et al.*, 2011; Lakshmipathy *et al.*, 2007). miRNA–mRNA pairs can also be plotted in a time series line plot. This plot can be scaled and interpolated over a spline (Fig. 1H). Two types of regression analysis can also be performed. First, a linear model is generated from a selected gene (mRNA or miRNA) and any number of its predicted binding partners. The combination of miRNA–mRNA interactions is left for the user to define. The longitudinal behavior of the selected gene is predicted based on the binding partners used in the linear model. The predicted simulation and the gene data are plotted along with the *R*^2^ value and *P*-value (Fig. 1I). This type of regression prediction is useful in cases where a mRNA is targeted by multiple miRNAs or if a miRNA targets multiple mRNAs. Next, a linear model can be created from a single miRNA–mRNA pair. The odds-ratio is calculated from the regression coefficient. This measures the likelihood of one gene influencing the behavior of another gene and has previously been used as a metric to determine miRNA–mRNA relationships (Jayaswal *et al.*, 2009). 95% confidence intervals are calculated which give a range where there is a 95% certainty of the mean of the data being within the range (Fig. 1J) (Szumilas, 2010). Selecting a miRNA–mRNA pair to investigate can be made easier by plotting a heatmap which orders the interacting pairs by descending correlation (Supplementary Figure S2). Statistics generated from correlation and regression analyses may be overestimations if too few time points are found within the input data. Thus, the tool will generate an error if fewer than three time points are detected and warnings if fewer than five time points are detected.

### 3.5 Output of the *TimiRGeN* package and exportation of data from *R*

*TimiRGeN* is an open-ended tool that exports to networking software *PathVisio* and *Cytoscape* for further *in silico* analysis. The *TimiRGeN R* package produces two data files for upload onto *PathVisio*. A file which includes a single result type, e.g. Log2FC, from each time point and gene IDs. This can be uploaded into *PathVisio* to show how the genes in a signaling pathway of interest change over the time course. Also a file which contains all filtered miRNAs can be uploaded into *PathVisio*. The second file requires the user to install the *MAPPbuilder* app in *PathVisio* (Kutmon *et al.*, 2015). With this, changes over time in a miRNA integrated signaling network of interest can be visualized to show how the miRNAs may be influencing the signaling pathway. This type of display is ideal for bottom-up GRN construction (Supplementary Figure S3). Filtered miRNA–mRNA interactions can also be exported to *Cytoscape* for improved visualization and alternative analysis via *Cytoscape* apps (Smoot *et al.*, 2011). The enhanced graphics of *Cytoscape* are especially useful to visualize large numbers of miRNA–mRNA interactions (Supplementary Figure S4).

### 3.6 Data from nonpairwise DE

The FA kidney injury dataset had pairwise DE performed using the zero time point as the denominator. This type of pairwise analysis is recommended for time series datasets with <8 time points; however, longer time series datasets may be more suitable for DE without using the pairwise approach e.g. over a cubic spline, *maSigPro* or the *LRT* method with *DESeq2* (Conesa *et al.*, 2006; Spies *et al.*, 2019). In these cases, users are recommended to filter out significantly differentially expressed genes from averaged count or expression data, and to use this as input for *TimiRGeN*. Pathway enrichment can be used to identify the most enriched pathways from the whole time course or temporal clustering can first cluster genes based on temporal behavior. From here, genes can be sorted based on clusters, and then pathway enrichment can be used to identify enriched pathways from each temporal cluster. An alternative pipeline is shown in Supplementary Figure S5 and this is explained in Section 5 of the vignette.

### 3.7 Datasets with multiple interventions

More complex datasets may include interventions other than time. In these cases, *TimiRGeN* should be used for each individual time series and then the results can be contrasted between different interventions. This requires the same signaling pathway to be explored in each time series. As an example, the ‘Lung fibrosis’ pathway was analyzed in the FA and UUO datasets. A pipeline is shown in Supplementary Figure S6 and Section 6 of the vignette provides detail.

### 3.8 Hypothesis generation with *TimiRGeN*

**Fig. 2.**
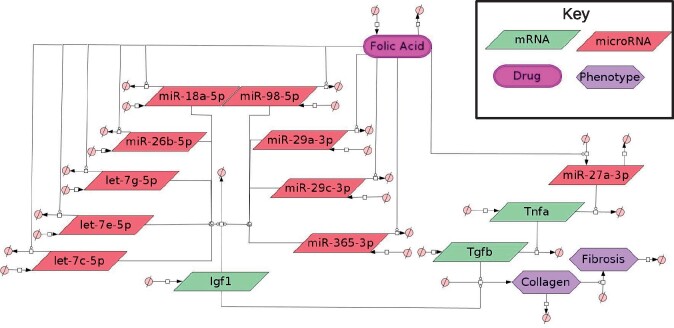
miRNAs influencing antifibrosis factor Tnfa and profibrosis factor Igf1: This GRN shows how FA may be downregulating *let-7c-5p*, *let-7e-5p*, *let-7g-5p*, *miR-18a-5p*, *miR-26b-5p*, *miR-29a-3p*, *miR-29c-3p*, *miR-365-3p* and *miR-98-5p*, which are all predicted to target profibrosis factor *Igf1*. Also this GRN indicates how FA may upregulate *miR-27a-3p*, which is predicted to target antifibrosis factor *Tnfa*. Reduction of *Tnfa* will increasing levels of profibrosis factor *Tgfb*

To demonstrate the tools ability to generate biologically relevant hypotheses, the FA mouse kidney injury dataset was analyzed with *TimiRGeN* (Fig. 1). Findings from the analysis were used to hypothesize how of FA can induce fibrosis. A GRN was constructed to formalize the hypotheses (Fig. 2). Investigation of these results can be used to ratify the miRNA–mRNA interactions predicted by *TimiRGeN* and make a stronger case for experimental validation. FA injection is known to cause acute injury conditions in the kidneys, resulting in a reversible chronic kidney disease (CKD) like condition (Craciun *et al.*, 2016; Pellegrini *et al.*, 2016). During the 14 day time course, a number of different processes occur, such as inflammatory response, scar tissue forming, wound healing, cytokine activity (Leask and Abraham, 2004). *TimiRGeN* analysis highlights several of these processes and GRNs were generated to represent how miRNAs may be influencing fibrosis factors (Fig. 2) and scar tissue forming by collagen synthesis (Supplementary Figures S7–S10). The GRN presented in Figure 2 indicates that *Igf1* acts as a miRNA sponge. Many of the presented miRNA-*Igf1* interactions have been reported, including *miR-18a*, *miR-98*, *miR-365* and *miR-26b* (Hu *et al.*, 2013; Liu *et al.*, 2016, 2017; Sun *et al.*, 2019). *let-7c-5p* has been reported to target *Igf1*, and *TimiRGeN* predicted other let-7 family genes *let-7e-5p* and *let-7g-5p* also target *Igf1* (Liu *et al.*, 2018). Finally, miR29 family members are predicted to target *Igf1*, and research indicates that *Igf1* is a *miR-29* family sponge (Gao *et al.*, 2016). It is unknown why *Igf1* may be a miRNA sponge, but *Igf1* is known to induce collagen production, which contributes to kidney fibrosis and CKD (Hung *et al.*, 2013). Exploration of *Igf1* as a miRNA sponge in kidney injury conditions could be beneficial for therapeutics for CKD. Overall, this case study highlights that the *TimiRGeN R* package can be used to identify biologically relevant miRNA–mRNA interactions from potentially tens-of-thousands of possible miRNA–mRNA interactions. The ability to reduce the volume of big multiomic data is an important feature of *TimiRGeN* and one which could lead to making miRNA research easier and faster for users. Further analysis on a breast cancer dataset is also found in Supplementary Figures S11–S16.

## 4 Conclusion

As recognized in Bar-Joseph *et al.* (2012), generation of complex transcriptomic datasets will continue, so computational biologists will need more sophisticated and up-to-date software to analyze these datasets (Bar-Joseph *et al.*, 2012). We have presented a novel *R/Bioconductor* package which aims to help researchers find direction when working with large longitudinal multiomic datasets. Overall, *TimiRGeN* is a useful new tool which could become a part of miRNA–mRNA data analysis pipelines.

## Funding

K.P., I.M.C. and D.Y. are supported by the Dunhill Medical Trust [R476/0516]. D.P.S. is supported by Novo Nordisk Fonden Challenge Programme: Harnessing the Power of Big Data to Address the Societal Challenge of Aging [NNF17OC0027812]. C.P., D.Y. and S.C. by the MRC and Versus Arthritis as part of the Medical Research Council Versus Arthritis Centre for Integrated Research into Musculoskeletal Ageing (CIMA) [MR/R502182/1].

*Conflict of Interest*: none declared.

## Supplementary Material

btab377_Supplementary_Data
