# Supplementary material for: TimiRGeN: R/Bioconductor package for time series microRNA–mRNA integration and analysis
Source: Bioinformatics. 2021 May 16;37(20):3604–9. doi: 10.1093/bioinformatics/btab377 (PMC8545325; doi:10.1093/bioinformatics/btab377)
Supplement: btab377_Supplementary_Data [file btab377_supplementary_data.zip › bioinf-2021-0127.R2_SupplementaryData.pdf]

Supplementary data: TimiRGeN: R/ Bioconductor package for time series microRNA-mRNA integration and analysis

Supplementary figures from analysis of the Lung fibrosis pathway

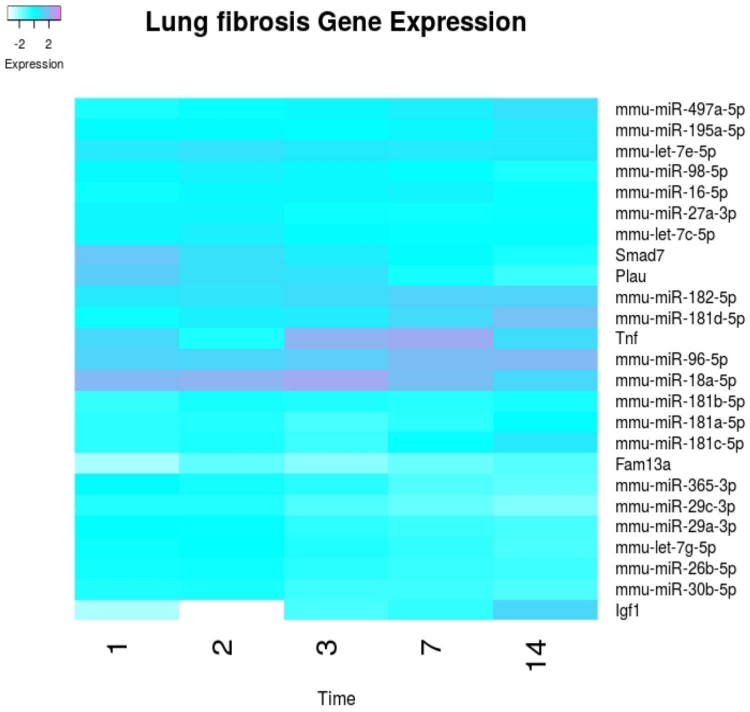

Supplementary Fig. 1: Heatmap showing gene expression of miRNAs and mRNAs. Selection of genes is the result of filtering for miRNA-mRNA interactions which affect the "Lung fibrosis" pathway. This plot is compatible with the dendrogram shown in Fig.1E from the main paper.

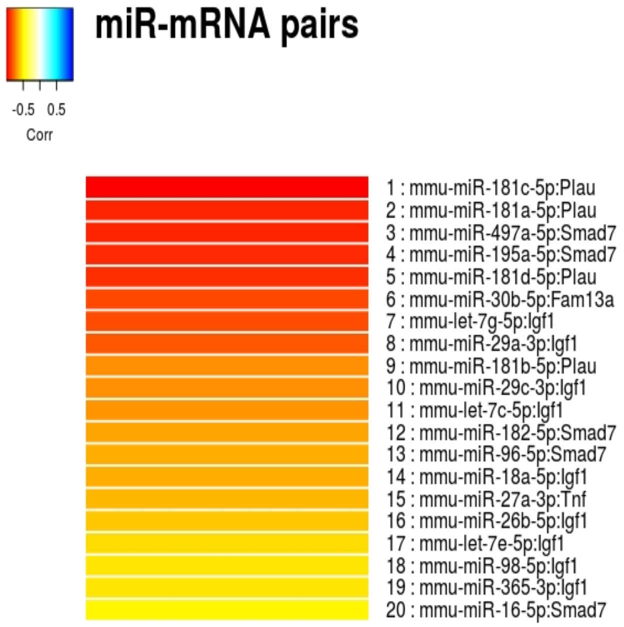

Supplementary Fig. 2: Heatmap showing the most negatively correlated miRNA-mRNA pairs in ascending order of correlation. These are miRNA-mRNA pairs filtered from the FA kidney injury analysis (Fig.1 from main paper). Interaction pairs are ordered and numbered here to ease the selection of a miRNA-mRNA pair for cross-correlation analysis, correlation plotting and regression analysis.

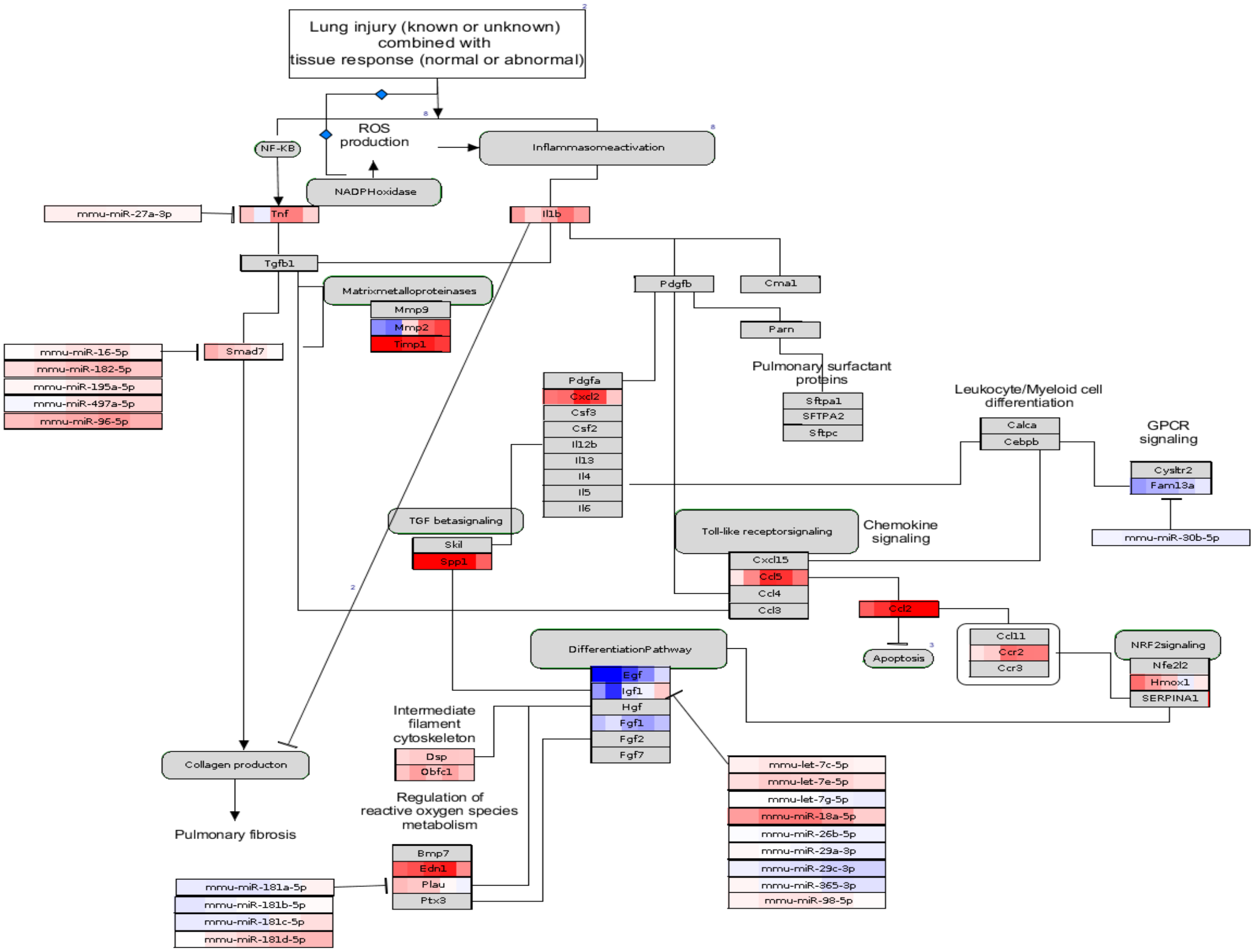

Supplementary Fig. 3: Modified "Lung fibrosis" wikipathway shown in *PathVisio*. This pathway has had sections removed to make viewing easier. miRNAs and dynamic information have been added from data files generated by *TimiRGeN*. Genes found in the dynamic information are colour coded based on Log2FC at each pairwise DE comparison. The range is -5 to 5. Negative fold changes are blue and positive fold changes are red.

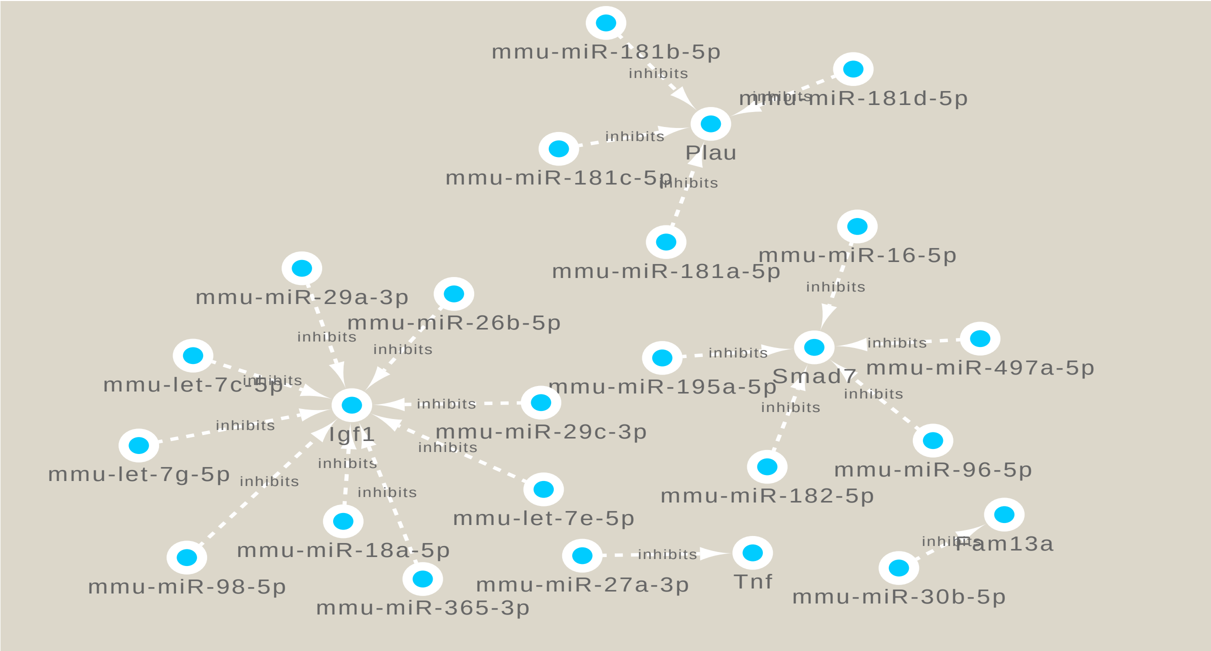

Supplementary Fig. 4: Filtered miRNA-mRNA network exported to *Cytoscape*.

Alternative pipelines

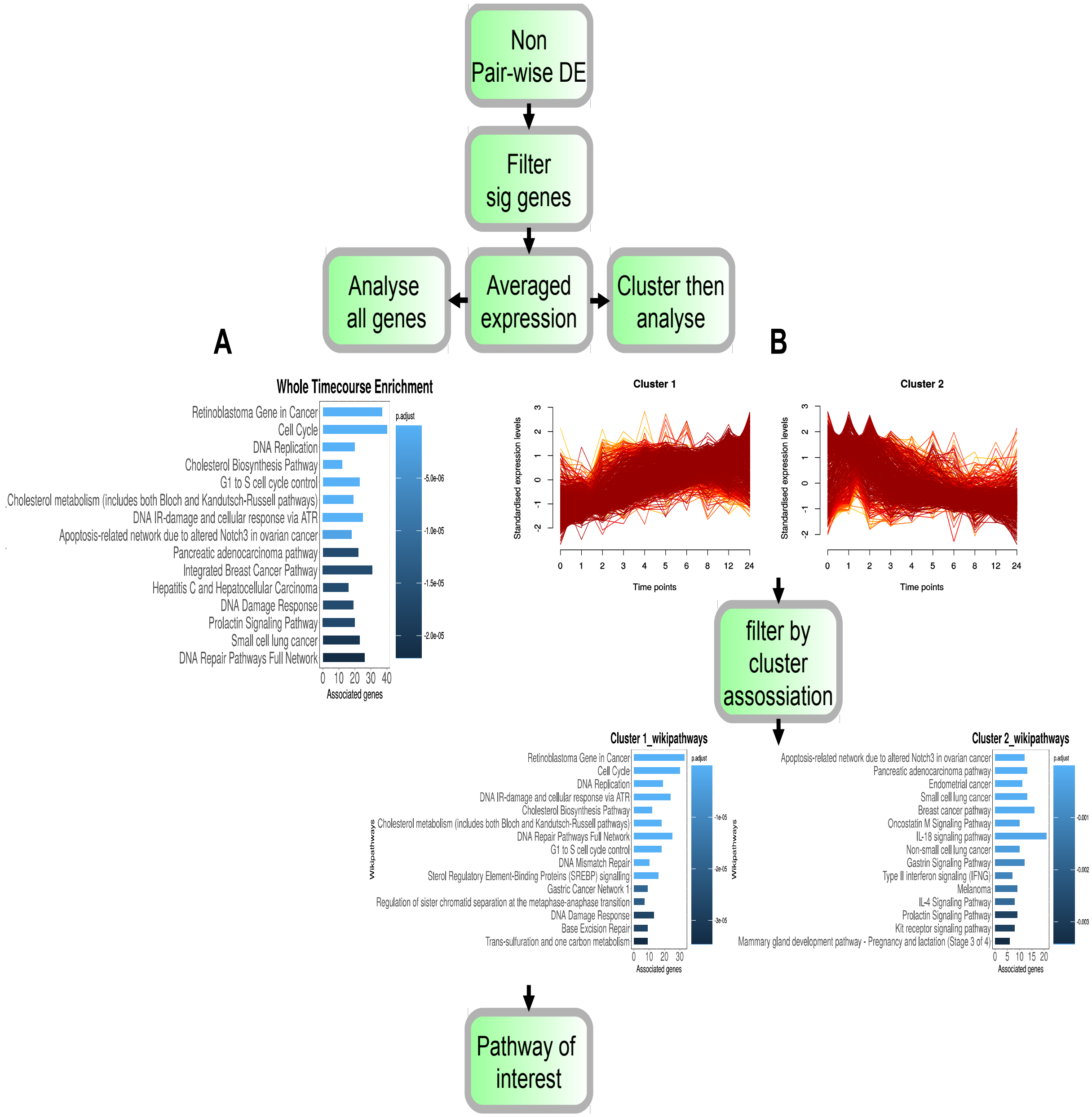

Supplementary Fig. 5: Alternative *TimiRGeN* pipeline for non-pairwise DE. Here averaged expression values of significantly DE genes can be taken forward to identify pathways of interest. A) all genes can be functionally analysed or B) genes can be clustered first, and then functionally analysed. After clustering, genes can be filtered by their association to a particular cluster. The threshold of this association is user defined.

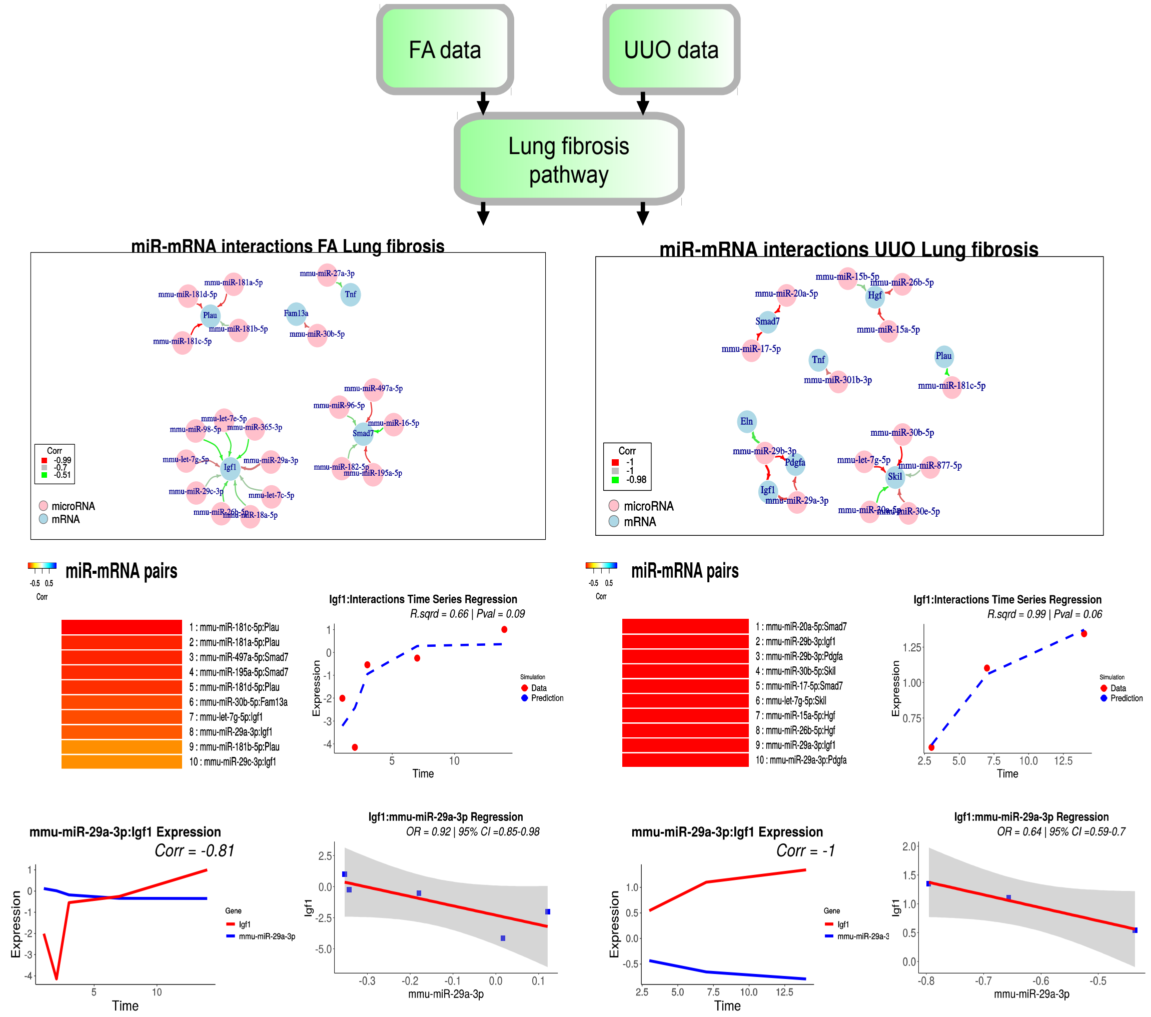

Supplementary Fig. 6: Expanded *TimiRGeN* pipeline for multivariate datasets. Here the FA and the UUO kidney injury datasets are compared using longitudinal pair analysis methods, within the context of the Lung fibrosis pathway. miRNA-mRNA interactions from the FA dataset have a negative correlation of < -0.5 and found in at least two databases. miRNA-mRNA interactions from the UUO dataset have a negative correlation of < -0.98 and found in at least two databases. Heatmaps show that *mmu-miR-29a-3p-Igf1* was found as the 8th and 9th most negatively regulated pair in the FA data and UUO data, respectively. Comparing the correlation and regression prediction plots, shows that the miRNA-mRNA interaction has a lower negative Pearson correlation score, a higher R^2 and lower p value in the UUO dataset. Regression analysis shows the odds-ratio is higher in the FA dataset, also the CI range is larger in the FA dataset.

From this analysis (S Fig.6) *mmu-miR-29a-3p-Igf1* may be an active miRNA-mRNA pair which regulates the "Lung fibrosis" pathway after FA or UUO induced kidney injury in mice. However, it seems this interactions may affect the "Lung fibrosis" pathway slightly more in the context of UUO induced kidney injury. However, because only three time points are present in the UUO dataset, the statistics may be artificially inflated. Thus S Fig.6 only serves to show an example of how *TimiRGeN* can be used to analyse multivariate datasets, rather than establishing any biological rational seen in the example.

Constructing a GRN for collagen synthesis from a mouse kidney injury dataset

The Folic acid (FA) mouse kidney injury dataset was processed and analysed, as described in the methods section of the main paper. Folic acid injections in kidneys lead to acute kidney injury conditions which resulted in phenotypes such as fibrosis, nephropathy and chronic kidney disease (CKD). This dataset was further explored by the *TimiRGeN* R package to identify mechanistic pathways of interest and to predict miRNA-mRNA interactions which may be influencing these pathways over the 14 day time course. The hypotheses generated from this process are formalised as (GRNs) gene regulatory networks to provide a useful framework for further work.

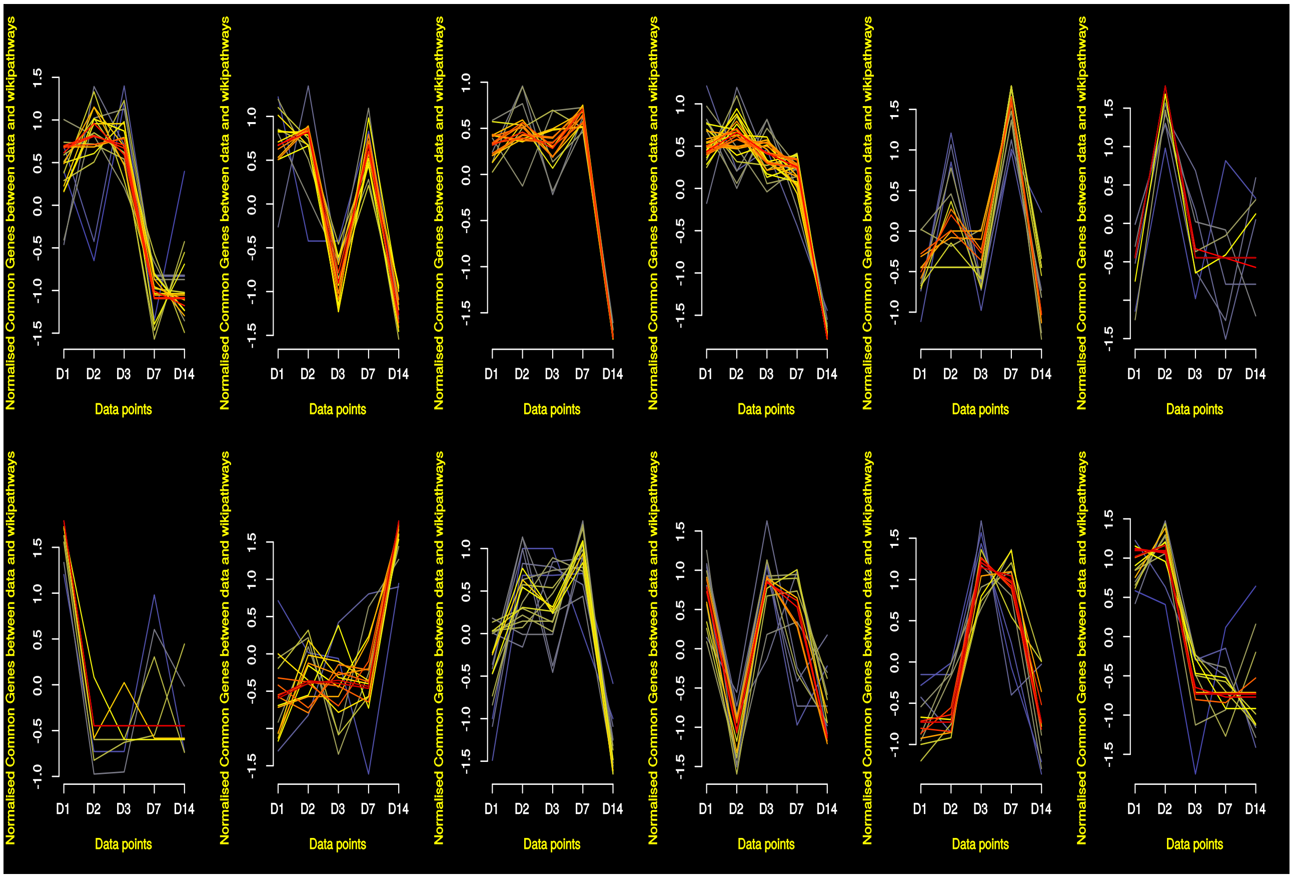

Supplementary Fig. 7: Output from the *quickFuzz* function of the *TimiRGeN* R package. 12 temporal profiles represent changes in pathways. Colour of the lines indicate the fitness score of a pathway to a cluster, in descending order of fitness is: red, orange, yellow, blue.

Temporal fuzzy clustering (S Fig.7) provides a global view of how the number of significantly differentially expressed (DE) genes within pathways change over the time course of the FA kidney injury dataset. Many of the clusters had interesting temporal behaviours, such as cluster 11. Pathways here have a sharp increase in the number of associated significantly DE genes from day 2 and this increase in associated genes lasts until day 7. Then the number of associated genes declines to normal levels at day 14. Pathways with a high fitness to cluster 11 are having some major activity during days 2-7 of the kidney injury model dataset. For example, the "Inflammatory Response Pathway" (IRP) (WP458) has a high fitness score to cluster 11. IRP was interesting because fur-

ther analysis with the *TimiRGeN* R package predicts 14 miRNA-mRNA interactions affecting the IRP during the kidney injury model (S Fig.8). The 14 interactions are filtered out because they have a negative average correlation and are found in at least two databases.

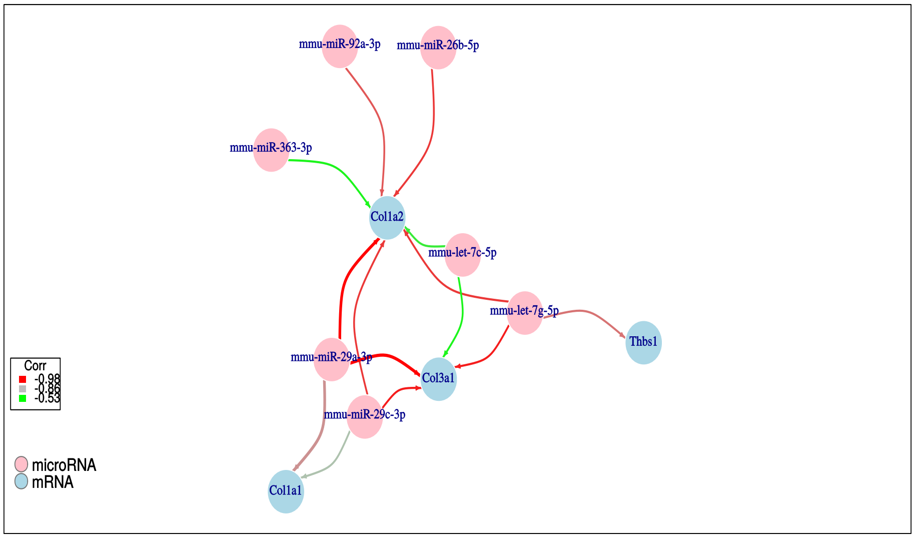

Supplementary Fig. 8: Output from *quickNet* function of *TimiRGeN* illustrates 14 predicted miRNA-mRNA interactions in this system. Pink and blue circles respectively represent miRNAs and mRNAs. Edge colour reflect the average correlation between the Log2FC values of the miRNAs and mRNAs, over the time course.

Results from *makeMapp* and *makeDynamic* functions are uploaded onto *PathVisio* using the instructions in 'Pathvisio_GRN_guide.pdf' (see link below). In *PathVisio* the miRNA and dynamic data are added to the IRP network (S Fig.9). Analysing the network indicates most of the miRNAs are affecting collagen genes. Using *CellDesigner* a Gene Regulatory Network (GRN) (S Fig.10) was constructed to formalise this hypothesis.

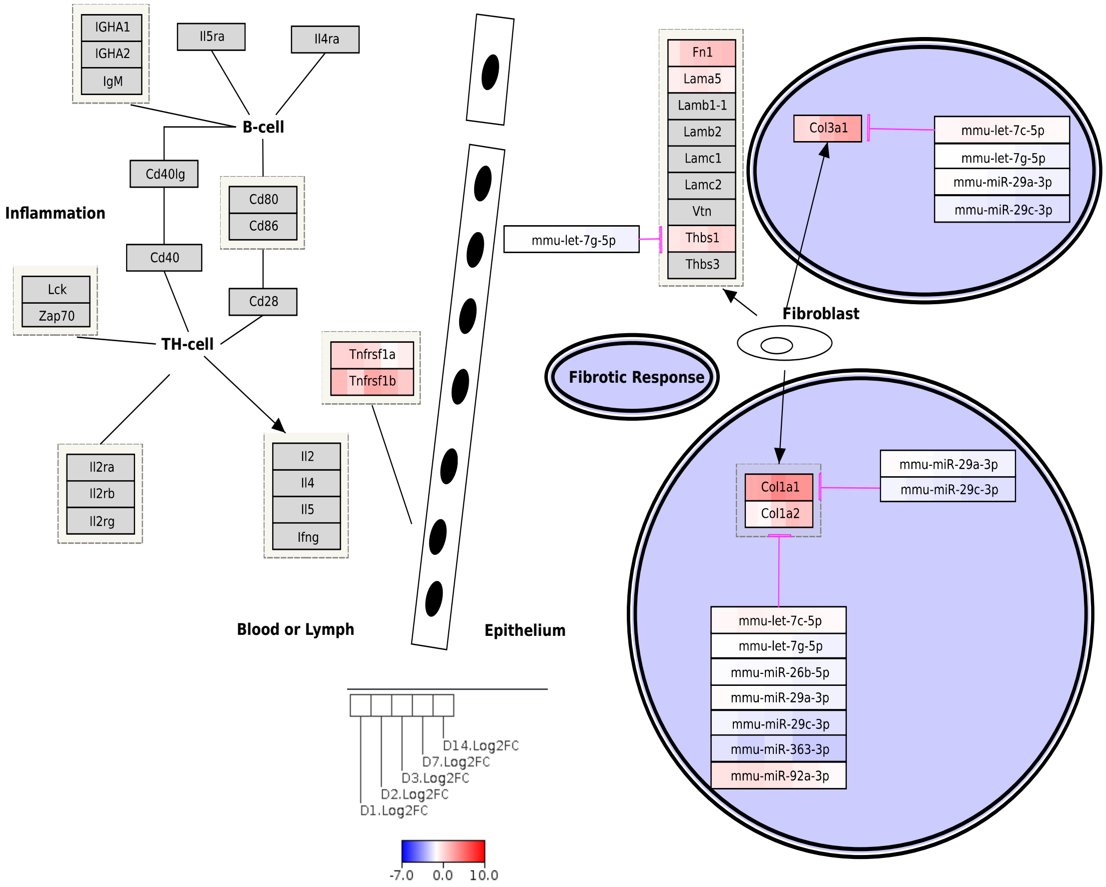

Supplementary Fig. 9: Inflammatory Response Pathway network combined with miRNAs and dynamic data from *TimiRGeN*. Blue circles are components which which have been selected for GRN construction. The key shows how the time series data is distributed in chronological order and the Log2FC values range from -7 to +10, where negative fold changes are blue, and positive fold changes are red. Lighter coloured/ white shadings indicate little fold change. Grey shaded boxes are genes not in the dataset. miRNA interaction edges are coloured pink.

The input for the collagen synthesis GRN is folic acid, which induces a fibrotic response process where increased immune cell activity and repair function leads to an increased rate of structural collagen production (Wen *et al.*, 2012; Stallons *et al.*, 2014). Transcription of genes such as *Col1a1*, *Col1a2* and *Col3a1* contribute to the accumulation of structural extracellular fibers. This will decrease the elasticity of surrounding tissues (Genovese *et al.*, 2014). RNAseq data shows that each of the collagen mRNAs increase in abundance over time, in contrast several miRNAs, predicted to target the collagen mRNAs, decrease in abundance. Using the *TimiRGeN* R package *miR-29a-3p* and *miR-29c-3p* are predicted to target each of the three collagen mRNAs. Several publications experimentally show the miR29 family play an important role in the regulation of *Col1a1* and *Col3a1* and that increased levels of miR29 family members, leads to a reduction in fibrosis (Wang *et al.*, 2019; Kriegel *et al.*, 2012; Broen *et al.*, 2014). Furthermore, our predictions indicate both *miR-29a-5p* and *miR-29c-5p* regulate *Col1a2*, which has not been explored in renal models.

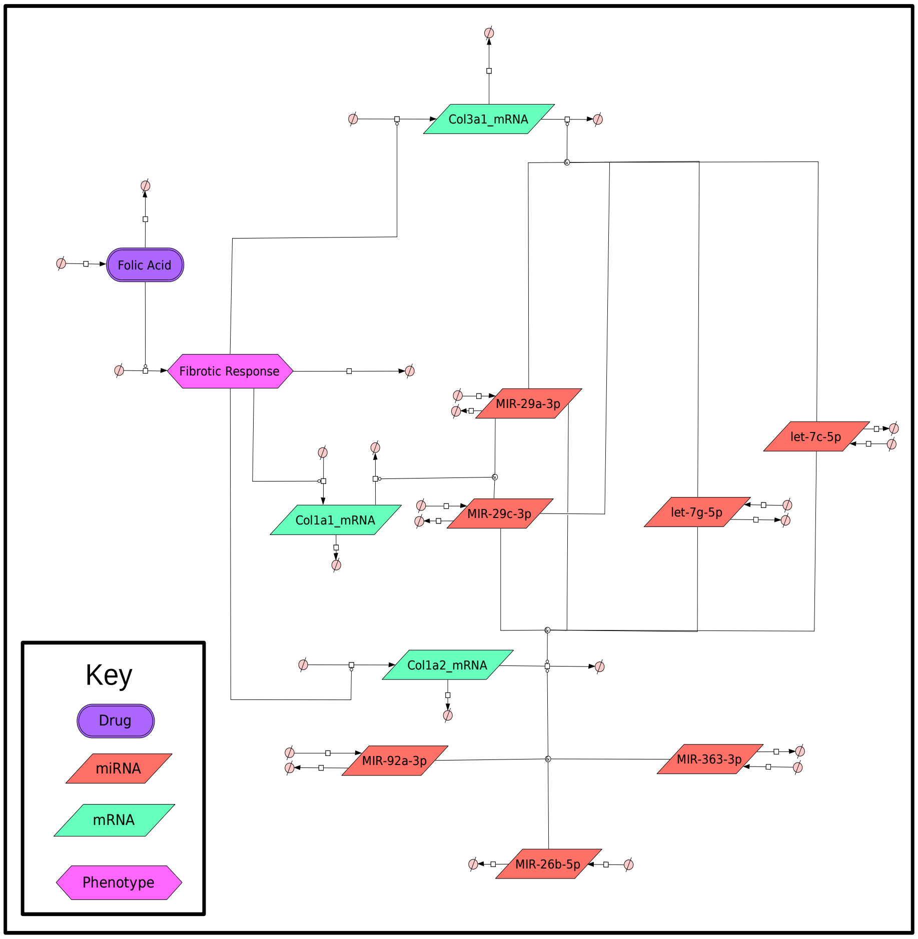

Supplementary Fig. 10: Collagen synthesis GRN which shows miRNA-mRNA interactions centred around collagens (*Col1a1, Col1a2, Col3a1*). *miR-29a-3p*, *miR-29c-3p* have widespread affects on all the collagens, *let-7c-5p* and *let-7g-5p* regulate *Col1a2 and Col3a1*, and *miR-26b-5p*, *miR-92-3p* and *miR-363-3p* regulate *Col1a2*.

We also predict Let7 family members to regulate structural collagen genes during kidney injury. Results from *TimiRGeN* analysis suggest *let-7g-5p* and *let-7c-5p* target both *Co1a2* and *Col3a1* mRNAs, and there is experimental evidence of *let-7d* directly targeting *Col3a1* mRNA (Su *et al.*, 2014). The Let7 family members share the majority of their seed sequence, strengthening the hypothesis of *let-7c-5p* and *let-7g-5p* also targeting *Col3a1* mRNA (Roush and Slack., 2008).

Results also indicate *miR-26b-5p*, *miR-92-3p* and *miR-363-3p* target *Col1a2* mRNA, though only *miR-26b* has experimental validation of targetting *Col1a2* mRNA (Tang *et al.*, 2017). We hypothesise that *miR-92a-3p* and *miR-363-3p* are novel targets of *Col1a2*, under kidney injury conditions. This GRN provides a guide for further work to test the predicted miRNA-mRNA interactions for their role in regulating fibrosis after kidney injury. This could lead to insights into miRNA based therapeutics for CKD.

Constructing a GRN which investigates the induction of myofibroblast activating factors in the tumour microenvironment using the *TimiRGeN* R package

A population of cancer-associated fibroblasts (CAFs) become activated to form myofibroblasts in the cancer stroma, where they induce tissue stiffness through extracellular matrix (ECM) remodelling (Liu *et al.*, 2019). Tissue stiffness promotes metastasis through breach of the basement membrane stimulating cancer cell migration, and through the induction of epithelial-mesenchymal-transition (EMT) cells which activates stem cell properties in cancer cells (Roche *et al.*, 2018). EMT cells are also a source of pro-fibrotic signals that induce fibroblast activation to form contractile myofibroblasts (Yao *et al.*, 2019). The importance of the ECM, and the role of tissue fibrosis and fibroblast activation in cancer has been extensively reviewed (Lu *et al.*, 2012; Hanahan *et al.*, 2011). The *TimiRGeN* R package is used here to investigate the induction of pro-fibrotic miRNA-mRNA interactions in the breast cancer tumour tissue environment.

Gene Expression Omnibus (GEO) and Array Express were searched for longitudinal miRNA-mRNA datasets for estrogen stimulation in breast cancer cells. The super-series GSE78169 was found in GEO Datasets, which contained 30 mRNA and 30 miRNA samples. MCF-7 breast cancer cell line samples were extracted prior to estradiol treatment, and 1-6, 8, 12 and 24 hours post treatment. Paired end mRNA and single end miRNA sequencing was done using Illumina HiSeq. A brief overview of the work flow used to analyse the data is shown in S Fig.11. The *TimiRGeN* R package was used for further exploration of the longitudinal miRNA-mRNA dataset. The package was used for functional analyses and to aid in the construction of a GRN. The results could be used for further computational work, mathematical modelling and as a resource to guide experimental work.

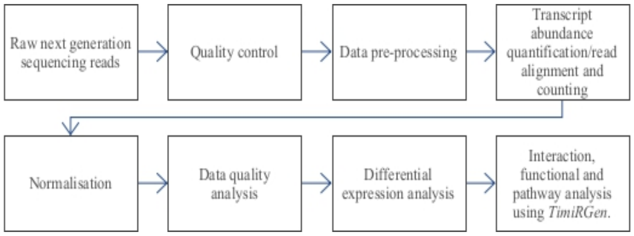

Supplementary Fig. 11: A brief overview of the workflow used to analyse raw data.

The *quickFuzz* function from *TimiRGeN*, uses a fuzzy clustering approach, to assess changes in behaviour of pathways across the time course, as shown in S Fig.12. Whereas S Fig.13 displays the results of pathway enrichment using *TimiRGeN*, which identified the "TGF-beta signalling pathway" (WP366) as significant in the early (1 hour) and late (24 hours) time point. This observation was consistent with the results from fuzzy clustering which showed that the "TGF-beta signalling pathway" fitted to a high degree in cluster 10. In this cluster, the genes in common between the data and the pathways change most at the 1 hour and 24 hour time points. This was expected as the role of TGF-beta in breast cancer is known to be paradoxical (Principe *et al.*, 2014). In early stages of carcinogenesis TGF-beta behaves as a tumour-suppressor by inducing apoptosis and cancer cell-cycle disruption. However, it promotes EMT and metastasis at the later stages (Heldin *et al.*, 2012). This prompted a further look into the "TGF-beta signalling pathway", as shown in S Fig.14, along with miRNA-mRNA interactions involved in this pathway which are shown in S Fig.15.

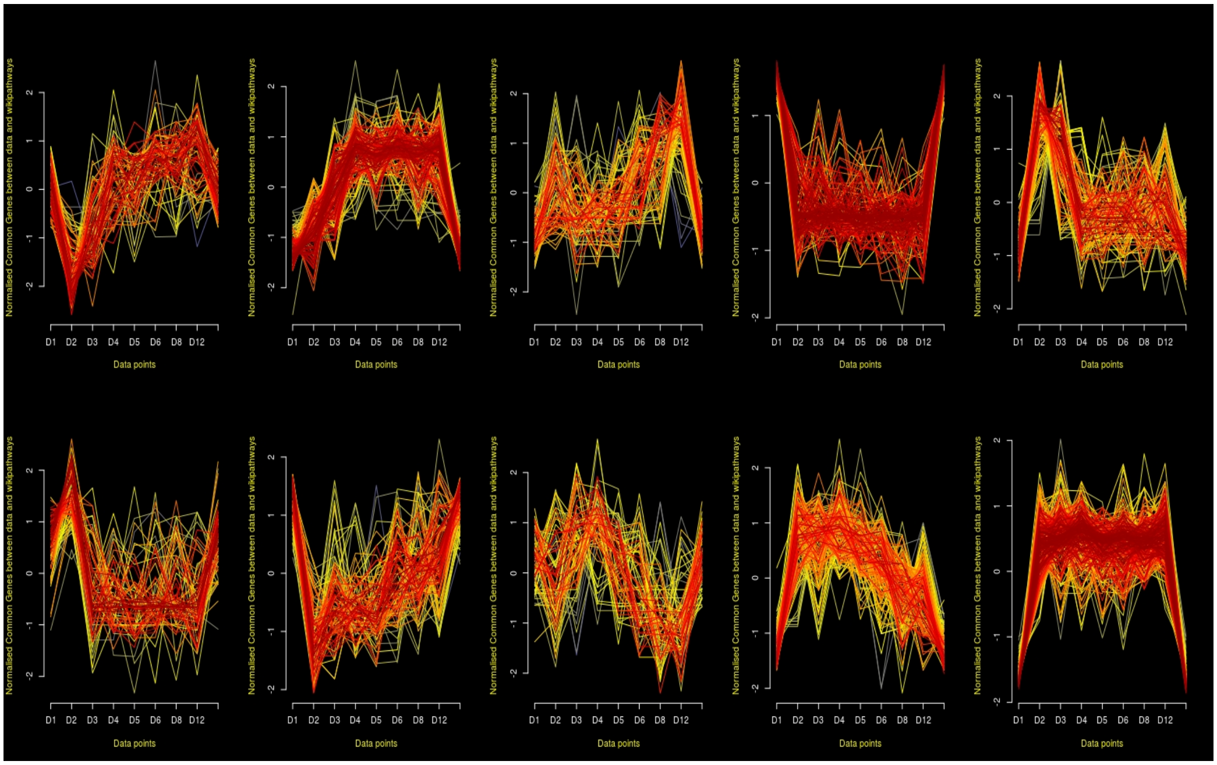

Supplementary Fig. 12: Fuzzy cluster plots were produced using the *quickFuzz* function from the *TimiRGeN* R package. The plot shows common genes between the data and the pathways changing over the time course (1-8, 12 and 24 hrs), and their behaviours over time is categorised into 10 clusters.

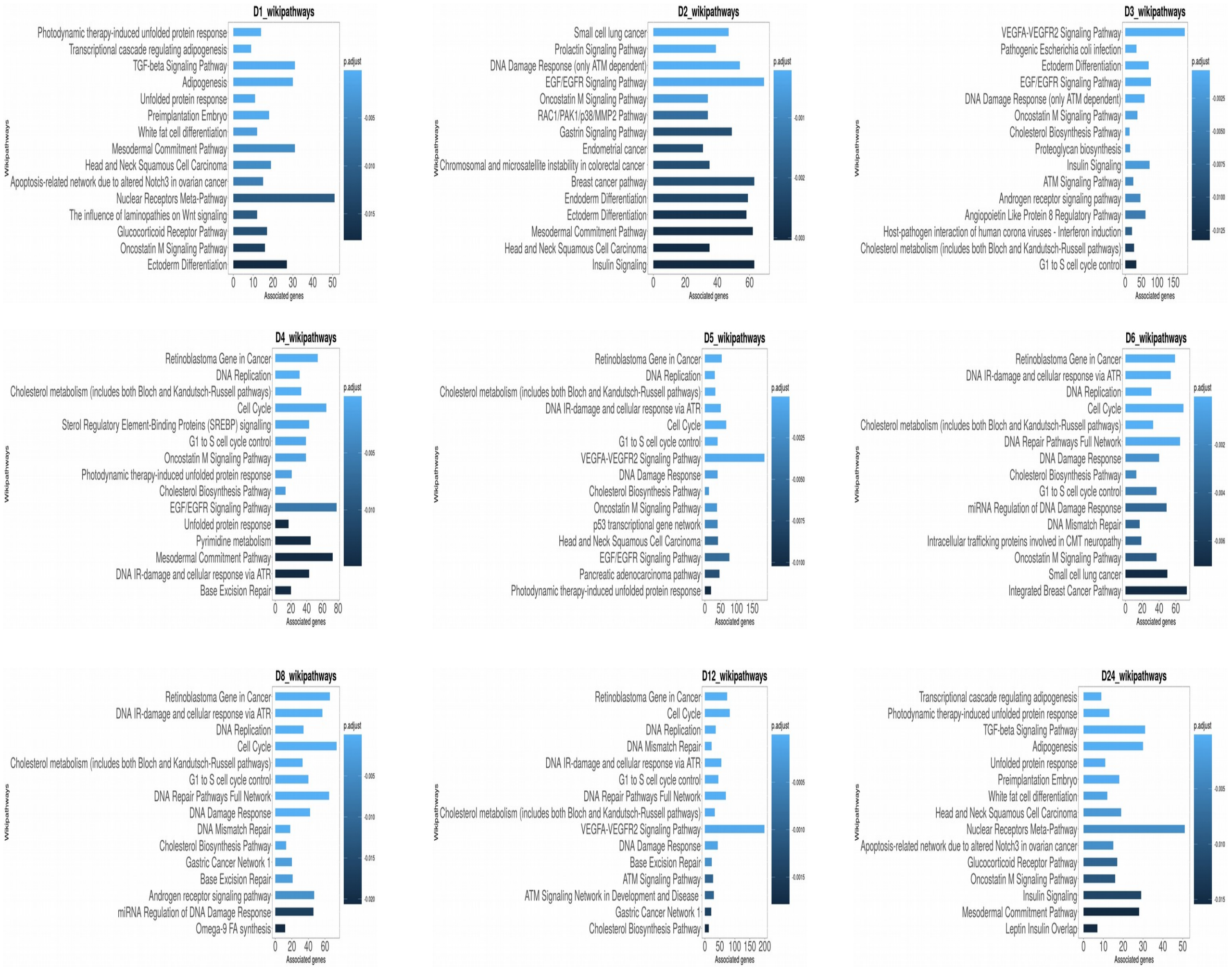

Supplementary Fig. 13: Enrichment plots, identifying which WikiPathways that are most enriched at each time point of the breast cancer study.

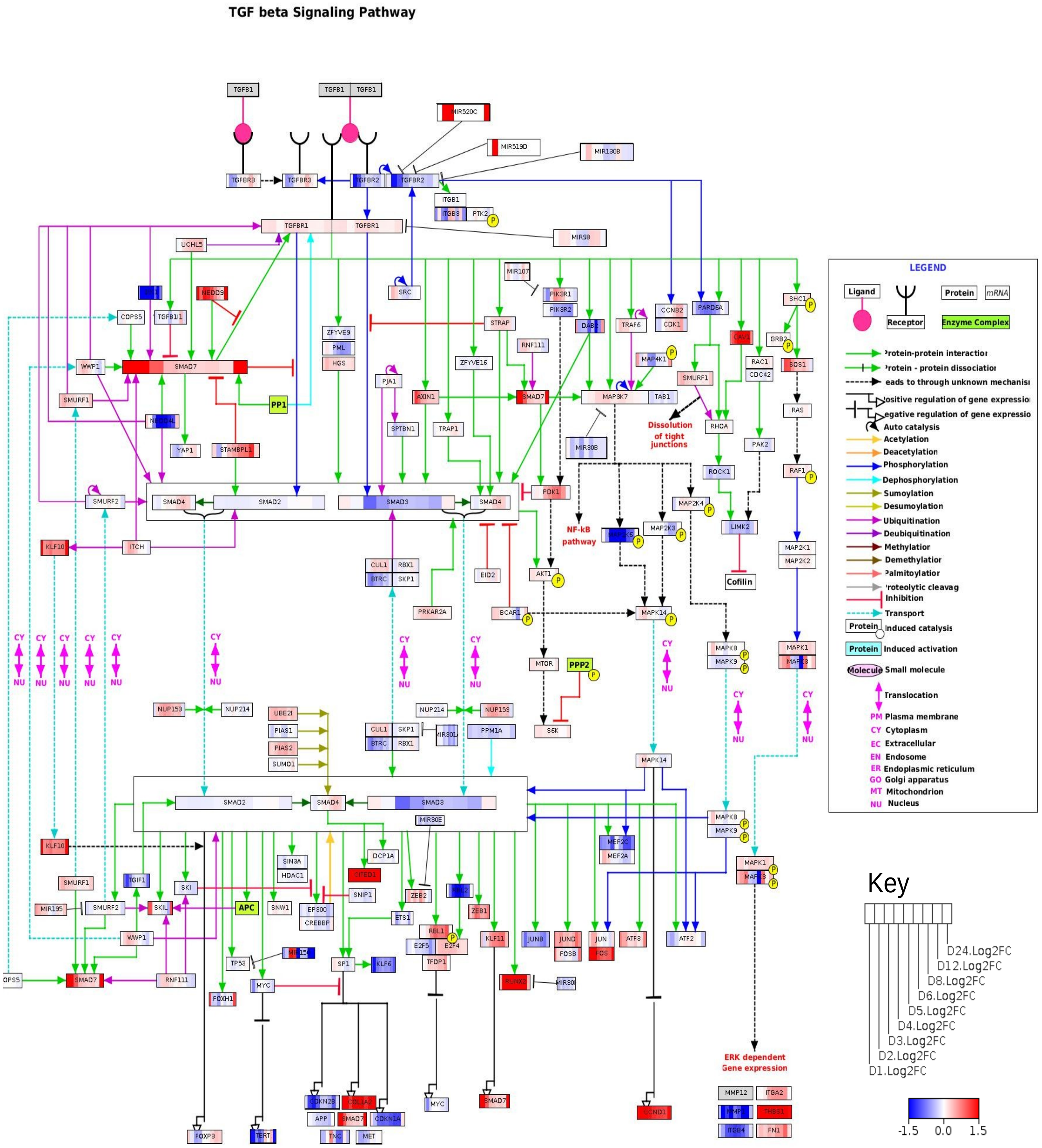

Supplementary Fig. 14: The "TGF-beta signalling pathway" along with miRNA and dynamic data from *TimiRGeN*. Data for the *PathVisio* network was created using the *makeDynamic* and *makeMapp* functions from *TimiRGeN*. Red and blue shadings indicate positive and negative Log2FC values, respectively. The range of the data is -1.5 to 1.5. Log2FC values are shown across the 9 time points in chronological order (1-6, 8, 12 and 24 hrs), as seen in the key. All miRNA-mRNA interactions shown have a maximum average correlation of -0.7.

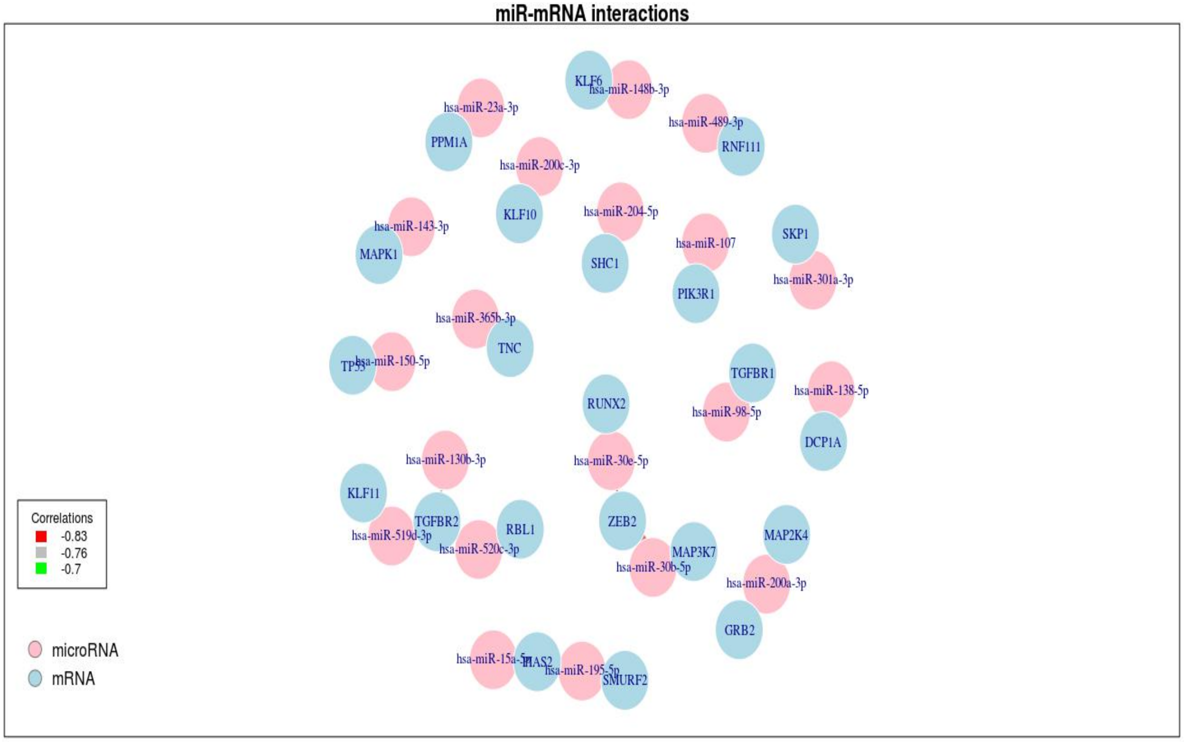

Supplementary Fig. 15: miRNA-mRNA interactions involved in the "TGF-b signalling pathway" shown using the *quickNet* function from *TimiRGeN*.

The GRN in S Fig.16 demonstrates mechanistic links between miRNA-mRNA interactions identified in the breast cancer dataset and how the miRNAs may influence the "TGF-beta signalling pathway". Literature evidence supports the presented regulatory links between *TGF-b1*, *Ski*, *TP53*, *Zeb2* and *Meox1/2*.

In the early stages of carcinogenesis, Smads and tumour suppressor *p53* act in conjunction, possibly through the formation of a complex, to mediate the transcription of *miR-30e*, which targets *Zeb2* mRNA for degradation (Elston and Inman., 2012; Laudato *et al.*, 2017). *ZEB2* is a transcription factor that induces *Meox1/2* repression although the exact mechanism is unknown. Since *Meox1/2* induces (alternatively spliced domain A) *EDA fibronectin* and *alpha-smooth muscle actin (a-SMA)* degradation. *ZEB2* degradation results in decreasing tissue stiffening (Zaha *et al.*, 2014; Wang *et al.*, 2017). Additionally, *ZEB2* not only induces *EMT*, but is also involved in the paracrine pro-fibrotic affects of EMT cells (Yao *et al.*, 2019). Therefore, the degradation of *Zeb2* by *miR-30e* in the early stages may be anti-tumorigenic.

However, in the later stages of carcinogenesis, pro-tumorigenic signalling activates by the reduction of *p53* activity by factors such as *miR-150-5p* (Liu and Di Wang., 2019). Paradoxically, TGF-beta/Smad signalling is also involved in the transcriptional activation of *Zeb2* (Cunnington *et al.*, 2014). This can result in the increase of *a-SMA* and pro-fibrotic *ECM* protein production. This subsequently leads to an increase fibroblast activation in the tumour stroma, leading to tumour matrix stiffness and fibrosis. Matrix stiffness correlates with high tumour invasion, and it is an

indicator of malignancy risk. For instance, high mammary density has been linked to increased risk of breast cancer (Boyd *et al.*, 2010). Increased stiffness acts in favour of cancer progression in several ways: it promotes cancer cell proliferation and invasion through the activation of the p38 MAPK pathway and promotes the increase in cancer stem cells (Provenzano *et al* 2009; Cordenonsi *et al.*, 2011). Stiff and linearised collagen fibres also promote metastasis through growth factors and integrin signalling-mediated cancer cell migration (Levental *et al.*, 2009).

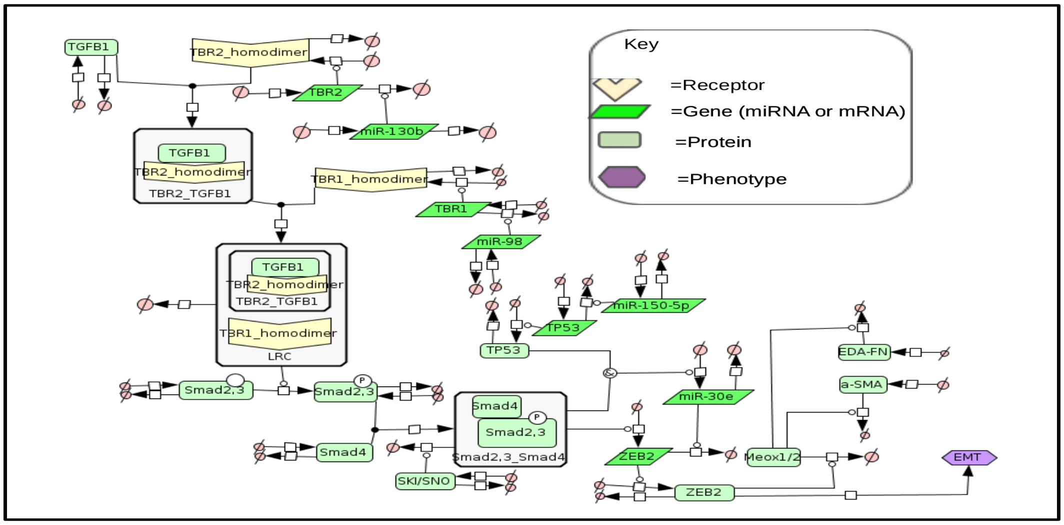

Supplementary Fig. 16: TGF-b induced tumour fibrosis GRN. This Network highlights links between *TGFB1, Ski, TP53, Zeb2* and *Meox1/2* during carcinogenesis. In this network, several miRNAs are predicted to regulate tumour fibrosis. Here *miR-130b* targets *TBR2*, *miR-98* targets *TBR1*, *miR-150-5p* targets *TP53* and *miRNA-30e* targets *ZEB2*.

Overall, the *TimiRGeN* R package has identified miRNA-mRNA interactions involved in the regulation of myofibroblasts in the tumour stroma through *TGF-b* and *ZEB2*. This includes miRNAs that regulate TGF-beta receptors, such as *miR-130b* and *miR-98*, since TGF-beta signalling is an important regulator of myofibroblast activity (Meng *et al.*, 2016). Although these interactions were expected, the *TimiRGeN* R package revealed a potential underlying mechanism behind TGF-beta mediated pro-tumorigenicity. That is, through the induction of tumour fibrosis mediated by the link between TGF-/Smad signalling, the pro-fibrotic *TP53/miR-30/ZEB2* axis and *EMT*. The interactions highlighted in S Fig.16 could be potential therapeutic targets to mitigate TGF-beta mediated pro-tumorigenicity and tumour fibrosis (Desgrosellier *et al.*, 2010).

Links to *TimiRGeN* resources

All scripts and data used in the main paper and supplementary data can be found here. A total of six runnable examples are here. 1) Complete investigation of the Lung fibrosis pathway using the FA kidney injury dataset. 2) Investigation of the inflammatory response pathway using a portion of the FA kidney injury dataset. 3) Analysing the TGF-beta signalling pathway using the 10 time point breast cancer dataset. This analysis is based on pairwise DE input. 4) Using the separate model of analysis on a microarray dataset. Here probes are used to adjust functional enrichment. 5) Averaged count data from the 10 time point breast cancer dataset is used as input. This analysis is based on non pairwise DE. 6) Multivariate analysis of the FA and UUO kidney injury datasets. https://github.com/Krutik6/TimiRGeN/issues/1

Vignette which displays multiple walk-through of *TimiRGeN* is found here. https://www.bioconductor.org/packages/release/bioc/vignettes/TimiRGeN/inst/doc/TimiRGeN_tutorial.html

Tutorial to use results *TimiRGeN* to create dynamic miRNA integrated networks in *PathVisio*. https://github.com/Krutik6/TimiRGeN/issues/2

Released package is available for download from Bioconductor. https://bioconductor.org/packages/TimiRGeN

References

Boyd, N. F. et al. (2010) Breast Tissue Composition and Susceptibility to Breast Cancer. J. Natl. Cancer Inst., 102, 1224-1237.

Broen, J. C. et al. (2014) The role of genetics and epigenetics in the pathogenesis of systemic sclerosis. Nat. Rev. Rheumatol., 10(11), 671-681.

Cordenonsi, M. et al. (2011) The Hippo Transducer TAZ Confers Cancer Stem Cell-Related Traits on Breast Cancer Cells, Cell, 147, 759-772.

Cunnington, R. H. et al. (2014) The Ski-Zeb2-Meox2 pathway provides a novel mechanism for regulation of the cardiac myofibroblast phenotype. J. Cell Sci.,127, 40-49.

Desgrosellier, J. S. and Cheresh, D. A. (2010) Integrins in cancer: biological implications and therapeutic opportunities. Nat. Rev. Cancer, 10, 9-22.

Elston, R. and Inman, G. J. (2012) Crosstalk between p53 and TGF- Signalling. J. Signal Transduct., 1-10.

Genovese, F. et al . (2014) The extracellular matrix in the kidney: a source of novel non-invasive biomarkers of kidney fibrosis? Fibrogenesis Tissue Repair, 7.

Hanahan, D. and Weinberg, R. A. (2011) Hallmarks of Cancer: The Next Generation. Cell, 144, 646-674.

Heldin, C. H. et al. (2012) Regulation of EMT by TGF in cancer. FEBS Lett., 586,1959-1970.

Kriegel, A. J. et al. (2012) The miR-29 family: genomics, cell biology, and relevance to renal and cardiovascular injury. Physiol. Genomics, 44(4), 237-244.

Laudato, S. et al. (2017) P53-induced miR-30e-5p inhibits colorectal cancer invasion and metastasis by targeting ITGA6 and ITGB1. Int. J. Cancer, 141, 1879-1890.

Levental, K. R. et al. (2009) Matrix Crosslinking Forces Tumor Progression by Enhancing Integrin Signaling. Cell, 139, 891-906.

Liu, F. and Di Wang, X. (2019) miR-150-5p represses TP53 tumor suppressor gene to promote proliferation of colon adenocarcinoma. Sci. Rep., 9.

Liu, T. et al. (2019) Cancer-associated fibroblasts: An emerging target of anti-cancer immunotherapy. J. Hematol. Oncol., 12, 86.

Lu, P. et al. (2012) The extracellular matrix: a dynamic niche in cancer progression. J. Cell Biol.,196, 395-406.

Meng, X. M. et al. (2016) TGF-B: The master regulator of fibrosis. Nat. Rev. Nephrol.,12, 325-338.

Principe, D. R. et al. (2014) TGF-B: duality of function between tumor prevention and carcinogenesis. J. Natl. Cancer I., 106.

Provenzano, P. P. et al. (2009) Matrix density-induced mechanoregulation of breast cell phenotype, signaling and gene expression through a FAK-ERK linkage. Oncogene, 28, 4326-4343.

Roche, J. (2018) The Epithelial-to-Mesenchymal Transition in Cancer. Cancers, 10.

Roush, S. and Slack, F. J. (2008) The let-7 family of microRNAs. Trends Cell Biol.,18(10), 505-516.

Stallons, L. J. et al. (2014) Suppressed mitochondrial biogenesis in folic acid-induced acute kidney injury and early fibrosis. Toxicol. Lett., 224, 326-332.

Su, B. et al. (2014) Let-7d suppresses growth, metastasis, and tumor macrophage infiltration in renal cell carcinoma by targeting COL3A1 and CCL7. Mol.Cancer, 13, 206.

Tang, C. M. et al. (2017) CircRNA 000203 enhances the expression of fibrosis-associated genes by derepressing targets of miR-26b-5p, Col1a2 and CTGF in cardiac fibroblasts. Sci. Rep., 7, 1-9.

Wang, J. P. and Hielscher, A. (2017) Fibronectin: How its aberrant expression in tumors may improve therapeutic targeting. J. Cancer, 8, 674-682.

Wang, R. et al. (2019) Long noncoding RNA DNM3OS promotes prostate stromal cells transformation via the miR-29a/29b/COL3A1 and miR-361/TGF1 axes. Aging, 11, 94429460.

Wen, X. et al. (2012) One dose of cyclosporine A is protective at initiation of folic acid-induced

acute kidney injury in mice. Nephrol. Dial. Transplant., 27,3100-3109.

Yao, L. et al. (2019) Paracrine signalling during ZEB1-mediated epithelialmesenchymal transition augments local myofibroblast differentiation in lung fibrosis. Cell Death Differ., 26, 943-957.

Zaha, D. C. (2014) Significance of immunohistochemistry in breast cancer. World J. Clin. Oncol., 5, 3820392.
