# Supplementary figures and images for: TimiRGeN: R/Bioconductor package for time series microRNA–mRNA integration and analysis
Source: Bioinformatics. 2021 May 16;37(20):3604–9. doi: 10.1093/bioinformatics/btab377 (PMC8545325; doi:10.1093/bioinformatics/btab377)

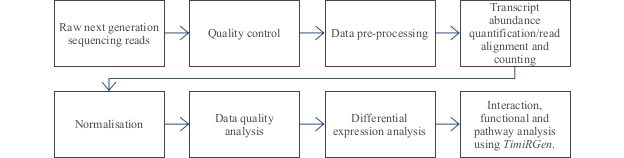

Supplement: btab377_Supplementary_Data [file btab377_supplementary_data.zip › SupplementaryFigure11_lowRes.jpg]

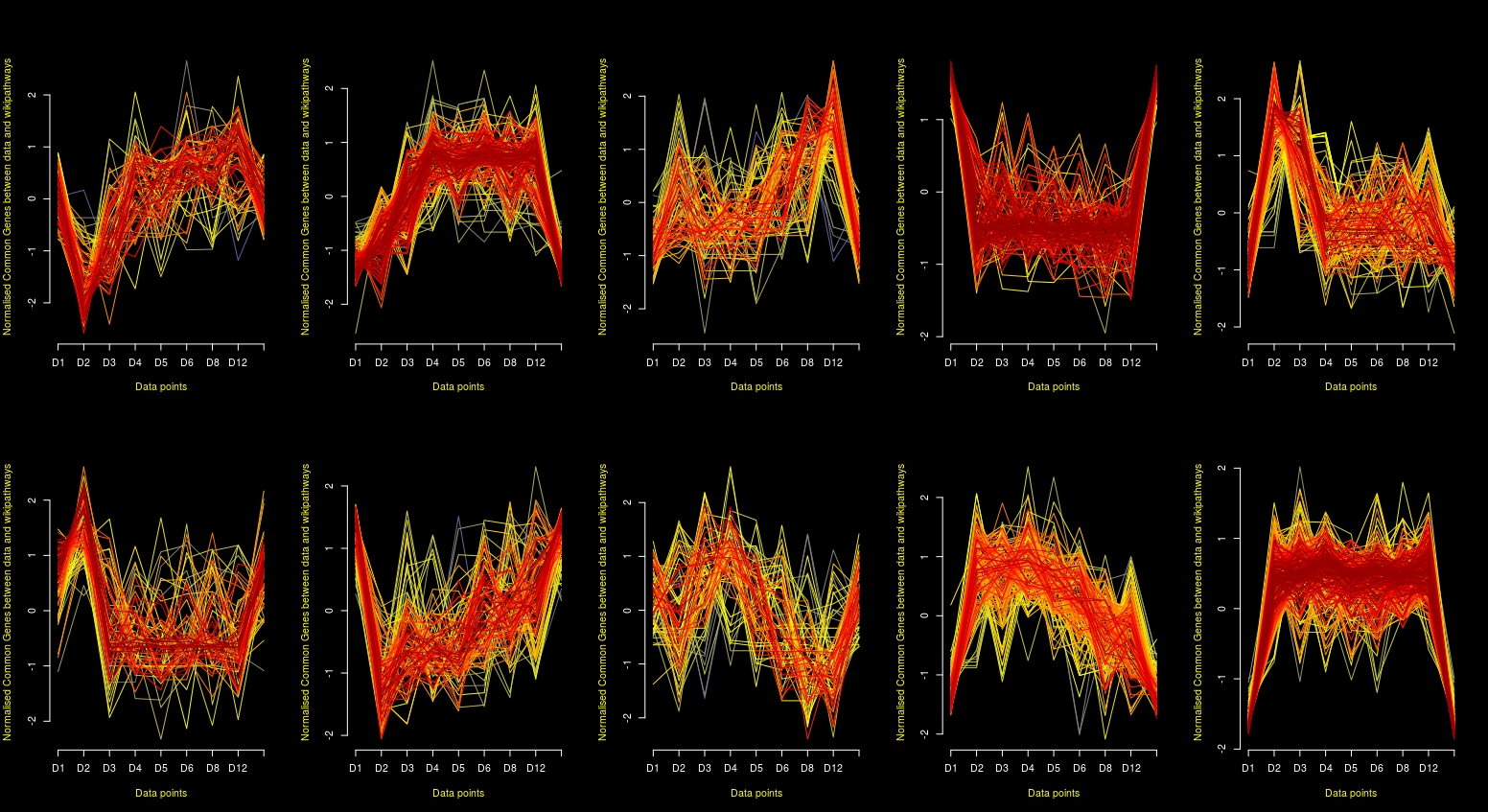

Supplement: btab377_Supplementary_Data [file btab377_supplementary_data.zip › SupplementaryFigure12_lowRes.jpeg]

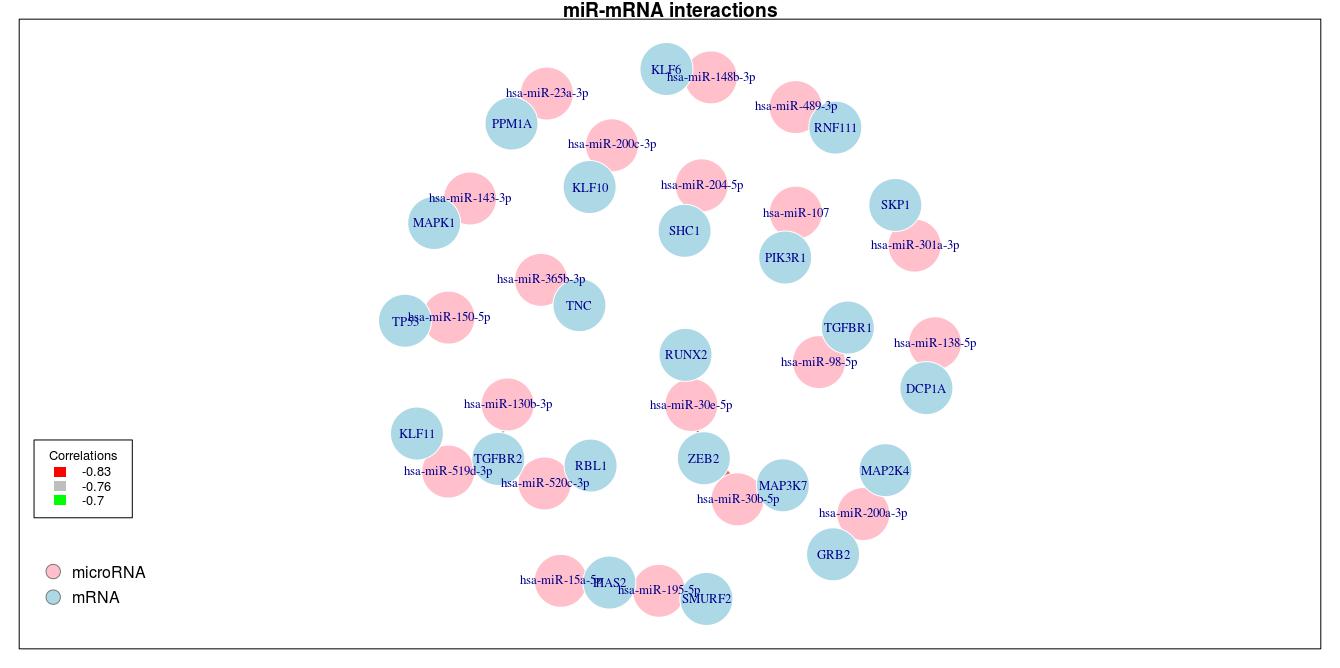

Supplement: btab377_Supplementary_Data [file btab377_supplementary_data.zip › SupplementaryFigure15_lowRes.jpg]
